# Beak and feather disease virus variant-dependent antibody reactivity in naturally infected psittacine birds

**DOI:** 10.1016/j.virusres.2026.199791

**Published:** 2026-08-16

**Authors:** Elora Kellerstrass, Monika Rinder

**Affiliations:** Clinic for Birds, Small Mammals, Reptiles and Ornamental Fish, Centre for Clinical Veterinary Medicine, University of Munich, 85764, Oberschleissheim, Germany

**Keywords:** Antigen variation, Beak and feather disease virus, Circovirus, ELISA, Recombinant capsid protein, Serology, Western blot

## Abstract

Beak and feather disease virus (BFDV, species *Circovirus parrot*) is the causative agent of psittacine beak and feather disease (PBFD), characterised primary by death of nestlings, feather loss and presumed immunosuppression in parrots worldwide. BFDV shows marked genetic heterogeneity, particularly in the open reading frame 2 (ORF2) encoding the capsid protein (Cap), but the impact of this diversity on antibody recognition has not been systematically assessed. In this study, we used seven recombinant Cap proteins derived from ORF2 variants selected to reflect the diversity of BFDV genotypes circulating in psittacine birds. N-terminally truncated, codon-optimised *cap* gene constructs were expressed in *Escherichia coli* and tested by semiquantitative Western blot and indirect ELISA using sera from eight naturally infected, BFDV-PCR-positive psittacine birds. Both assays revealed clear antigen-serum dependent differences in reactivity, with several sera failing to produce detectable signals against some Cap variants under the conditions used. These findings provide experimental evidence that amino acid variation in Cap can be associated with distinct antibody binding patterns in naturally infected birds. The observed heterogeneity in antigen recognition is likely to have direct implications for natural infections with BFDV, for the design and interpretation of BFDV serological assays and should be considered in future development of Cap-based vaccines.

## Highlights

• Diverse BFDV Cap genes result in antigenically diverse Cap proteins.
• Sera from naturally infected parrots differed in reactivity to variant Cap proteins.
• Single recombinant antigen did not capture serological responses of all sera.
• These findings were evident in both ELISA and Western Blot.
• BFDV Cap diversity has an impact on BFDV immunogenicity.

## 1 Introduction

Psittacine beak and feather disease (PBFD) is a highly contagious and often severe disease of psittacine birds worldwide. The causative agent is beak and feather disease virus (BFDV; species *Circovirus parrot*) which is classified within the genus *Circovirus* in the family *Circoviridae.* It is a small, non-enveloped virus with a single-stranded, circular DNA genome (ICTV, 2026b; Ritchie et al., 1989).

BFDV is a globally distributed pathogen that occurs at considerable prevalences in both wild species and birds kept in human care on multiple continents; a recent meta-analysis estimated a pooled global prevalence of 16.3 % (Varsani et al., 2011; Zhang et al., 2025). It poses a particular threat to small populations and endangered species, where even moderate prevalence can have disproportionate demographic effects (Das et al., 2020; Raidal et al., 2015). Infection has been documented in at least 78 psittacine species and all parrot species are considered susceptible to BFDV (Fogell et al., 2016; Morinha et al., 2020).

Clinically, affected birds may suffer from apathy, progressive feather dystrophy, feather loss or brittle and overgrown beaks as well as assumed BFDV-associated immunosuppression depending on their age and species (Latimer et al., 1990; Pass and Perry, 1984; Ritchie et al., 1989; Todd, 2000). Subclinical and latent BFDV infections appear to be common in both wild and captive psittacine populations, with virus detectable in clinically unremarkable birds and persisting in tissues for months to years, indicating long-term and potentially lifelong infection in at least some individuals (Blanch-Lázaro et al., 2021; Martens et al., 2019, 2020). Since there is no cure for PBFD and no vaccine is commercially available, early detection of infected birds and separation from susceptible stocks are key elements of PBFD control (Australian Government Department of Environment and Heritage, 2006).

At the molecular level, the approximately 2.0 kb circular genome of BFDV contains two major ambisense open reading frames (ORF1 and ORF2) with well-characterised functions: ORF1 encodes the replication-associated protein (Rep) and ORF2 encodes the capsid protein (Cap), which assembles into a T = 1 icosahedral capsid composed of 60 identical subunits (Bassami et al., 2001; Sarker et al., 2016a, 2016b). The Cap protein constitutes the major structural antigen of the virion, plays an important role in virus attachment and cell entry, and elicits virus-specific antibody responses (Johne et al., 2004; Nath et al., 2021; Shearer et al., 2008b). Consequently, recombinant Cap has been used as an antigen in serological assays and has also been evaluated as a subunit vaccine candidate in experimental vaccination and challenge studies (Bonne et al., 2009; Ndlovu et al., 2025).

An important characteristic of BFDV is its marked genetic diversity. Numerous genetically distinct genotypes have been described, forming several virus clades and subclades in recent phylogenetic analyses (Shah et al., 2023; Varsani et al., 2011). This diversity arises from a combination of point mutations, insertions/deletions within the genome and frequent recombination events (Bassami et al., 2001; Julian et al., 2013). There is growing evidence that some lineages show host association or host specificity (Bassami et al., 2001; Kloet and Kloet, 2004; Varsani et al., 2011). Both the *rep* and *cap* genes are subject to genetic variation, but variability is particularly pronounced in the ORF2, while the ORF1 is comparatively more conserved (Dolatyabi et al., 2022). *cap* sequence heterogeneity is evident at both the nucleotide and amino acid levels (Dolatyabi et al., 2022) suggesting that it may influence antigenic structure and epitope presentation as well as the development of a protective immunity.

Accurate diagnosis of BFDV infection is central to any control programme to detect infected birds for implementation of protective biosecurity measures. Since viral culture is not yet available for psittacine circoviruses, BFDV detection relies mainly on polymerase chain reaction (PCR)-based assays, which are widely used and mostly target the *rep* gene (Ogawa et al., 2005; Raidal et al., 2008; Ypelaar et al., 1999). However, when used on live birds, PCR can only identify those that are actively shedding viral DNA in the sampled material at the time of sampling. Asymptomatically infected carrier birds that are not shedding virus, or shed virus only intermittently at low levels, may therefore test negative and remain undetected, thereby posing a continued risk to presumably BFDV-free flocks (Martens et al., 2020). Furthermore, due to the high mutation rate of BFDV, there is always a risk of mutations occurring in critical 3′ ends of the PCR primer region, resulting in a failure of primer binding and thus a failure to detect the virus (false negative results).

Serological methods for detecting BFDV-specific antibodies, such as haemagglutination inhibition (HI) or enzyme-linked immunosorbent assays (ELISAs), can help to better assess the infection status of individual animals and monitor populations, but are at present not widely available. Both blocking ELISA (Shearer et al., 2009) and indirect ELISA (Das et al., 2026; Johne et al., 2004) are described, as well as HI as the serological gold standard (Raidal et al., 1993) but there are currently no investigations with the primary aim to examine how the genetic diversity in different BFDV genotypes influences the formation of specific antibodies in psittacine hosts or antibody detection in diagnostic tests. The BFDV antigenic variability raises the question of whether genotype-specific differences in Cap affect antibody binding, or whether a general cross-reactive immunity exists for divergent BFDV genotypes. This is not only relevant for the design and interpretation of serological assays in diagnostic settings but also for conceptualising potential vaccines and our understanding of natural host defence mechanisms and disease pathogenesis, although genetic variation cannot be assumed to directly translate into antigenic variability. However, there is currently a lack of empirical data if and how Cap variation effect antibody binding to different BFDV genotypes.

The research hypothesis for this study was that differences in antigenicity between genetically distinct BFDV variants influence antibody recognition in serological assays. We expressed a panel of recombinant Cap proteins representing genetically diverse BFDV genotypes and compared their relative serological reactivity with their underlying genetic diversity. It was expected that there would be variations in antibody levels between the sera of parrots naturally infected with BFDV. Therefore, the comparisons were conducted with the objective of ascertaining the reactivity of each bird to the various antigens. Quantitative comparisons of antibody levels between individual birds were not intended.

## 2 Materials and methods

### 2.1 Ethical statements

All samples used in this study complied with institutional and national guidelines for animal welfare. The study was approved by the Ethics Committee of the Veterinary Faculty, Ludwig-Maxililians-Universitaet Muenchen (file number 262–09–03–2021). Psittacine samples used in this work consisted exclusively of surplus material remaining from routine diagnostic procedures, which had been stored at −20 °C in the laboratory.

### 2.2 Primer design, cap gene amplification, structural visualization of capsid sequence variability

DNA solutions obtained from clinical samples of pet birds that had tested positive for BFDV in routine diagnostics by PCR targeting the rep gene (Ogawa et al., 2005; Ypelaar et al., 1999) were used in this study. Based on DNA sequence alignments of a representative number of BFDV genotypes included in the NCBI GenBank database (https://www.ncbi.nlm.nih.gov/genbank/), specific primers were designed to amplify the complete cap open reading frame (ORF2) of BFDV: forward primer cap 5´-CACCATGTGGGGCACCTCYAAC-3´, reverse primer cap 5`-CGAAAAGSTATTTGYYGTYTGAGTCTTTA-3` (degenerate positions according to IUPAC nucleotide ambiguity code). PCR amplification of ORF2 was performed using a high-fidelity DNA polymerase (SuperFi DNA polymerase, Thermo Fisher Scientific, Darmstadt, Germany) according to the manufacturer’s instructions. Reactions were run in a thermal cycler under the following conditions: initial denaturation at 95 °C for 5 min, followed by 35 cycles of 95 °C for 30 s, 60 °C for 30 s and 72 °C for 60 s, and a final extension step at 72 °C for 7 min.

*Cap* amplicons obtained from various BFDV-positive samples were gel-purified and subjected to Sanger sequencing (Eurofins Genomics, Ebersberg, Germany) in both directions. Consensus sequences were assembled and edited, then aligned using MUSCLE in MEGA version 11 (Tamura et al., 2021). The consensus sequences were subsequently compared with representative BFDV ORF2 sequences from clades Ia-If and IIa-IIb (Shah et al., 2023), retrieved from the NCBI GenBank nucleotide database. Seven *cap* amplicons covering the genetic range of current BFDV genotypes were selected for protein expression. Nucleotide sequences, which are available on the NCBI GenBank database under the accession numbers PPX789812-PX789818, were aligned using MUSCLE in MEGA11. The best-fit nucleotide substitution model was selected in MEGA11 using the Bayesian Information Criterion (BIC). Phylogenetic inference was performed using Maximum-likelihood under the TN93+G model (gamma-distributed rate heterogeneity, k = 5 discrete categories). Node support was assessed by bootstrap analysis with 1000 replicates. Sites containing gaps and missing data were treated using partial deletion with a site coverage cutoff of 95 %.

Pairwise distances were calculated using the p-distance method with uniform rates among sites. Sites containing gaps and missing data were eliminated by complete deletion. Pairwise percent identity was derived as (1 − p-distance) × 100.

To visualize structural conservation and variability, the amino acid sequences of Cap 1–7 were aligned together with the BFDV capsid sequence corresponding to the crystallographic reference structure (PDB: 5J36). The alignment was associated with the 5J36 pentamer in UCSF Chimera (version 1.19) (Pettersen et al., 2004) and rendered onto the molecular surface. Amino acid conservation across all aligned sequences was displayed using a colour gradient (green colour for fully conserved positions, increasingly variable positions shown along the gradient from blue to red). Surface views were generated for both the exterior and interior faces of the pentamer.

### 2.3 Plasmid construction, cloning, expression and purification of recombinant Cap protein

Recombinant expression of full-length BFDV Cap has previously been reported to be troubled by solubility and aggregation issues, which can be improved by truncation of the sequence and codon optimisation (Johne et al., 2004; Regnard et al., 2017). For recombinant protein expression, full-length cap sequences were truncated by 40 amino acids at the N-terminus and then codon-optimised for expression in *Escherichia coli* (GeneArt, Thermo Fisher scientific, Regensburg, Germany). The resulting synthetic gene DNA was then used for cloning and expression.

Codon-optimised, N-terminally truncated cap gene DNA were cloned into the pET100/D-TOPO expression vector (GeneArt, Thermo Fisher Scientific) to result in N-terminally His-tagged Cap proteins. The plasmids were transformed into *E. coli* Rosetta 2 (DE3) cells and plated onto LB agar containing ampicillin (100 µg/mL). After overnight incubation at 37 °C colonies were screened by PCR, and the PCR products of the expected size were Sanger-sequenced using forward and reverse primers within the vector to verify cap sequences and orientation of the insert. Single confirmed colonies were used to inoculate LB broth containing ampicillin and cultured overnight at 37 °C with shaking.

Pre-warmed LB broth (250 mL) supplemented with ampicillin was inoculated with 11 mL of the overnight culture and incubated at 37 °C with shaking until an optical density at 600 nm (OD_600) of 0.5–0.6 was reached. Protein expression was induced by adding isopropyl-β-D-thiogalactopyranoside (IPTG) to a final concentration of 1 mM, and cultures were incubated for a further 8 h at 27 °C with shaking. Cells were harvested by centrifugation (2200 x g, 20 min, 4 °C) and the resulting pellets were stored at −20 °C until further processing.

Cell pellets were resuspended in lysis buffer (375 mM NaCl, 48 mM potassium phosphate, 10 mM imidazole, 5 % (v/v) glycerol, 0.5 % (v/v) Triton X-100, pH 7.8) and lysed on ice by sonication. The lysates were clarified by centrifugation (13,000 x *g*, 10 min, 4 °C) to separate soluble and insoluble fractions. His-tagged Cap proteins were purified from the soluble fraction using Ni-NTA affinity chromatography (HisPur™ Ni-NTA spin columns, Thermo Fisher Scientific) following the manufacturer’s protocol. All equilibration, wash and elution buffers contained 10 % (v/v) glycerol to stabilise the protein. Eluted Cap proteins were stored at −20 °C.

### 2.4 SDS-PAGE, protein staining, Western blot analysis, protein assay, electron microscopy

Sodium dodecyl sulfate polyacrylamide gel electrophoresis (SDS-PAGE) was performed on all purified recombinant Cap proteins. Gels were stained with PageBlue™ protein staining solution (Thermo Fisher Scientific) to assess purity. For Western blotting, proteins were transferred onto nitrocellulose membranes using a standard protocol. Membranes were incubated overnight at 4 °C with a rabbit polyclonal anti-His tag antibody (Thermo Fisher Scientific; 1:1000 in PBST containing 2 % (w/v) skimmed milk powder). After washing with PBST, membranes were incubated for 30 min at room temperature with an HRP-conjugated anti-rabbit IgG secondary antibody (SIGMA-Aldrich, Steinheim, Germany; 1:2000 in PBST with 2 % (w/v) skimmed milk). Bound antibodies were visualised using an enhanced chemiluminescence (ECL) substrate (Amersham ECL prime, Cytiva, Marlborough, USA) according to the manufacturer’s instructions, and signals were recorded with a digital chemiluminescence imaging system (Celvin® S, biostep GmbH, Burkhardtsdorf, Germany).

Protein concentrations in elution fractions of all recombinant Cap preparations were determined by colorimetric protein assay (Bio-Rad, Feldkirchen, Germany) using bovine serum albumin (BSA) as the calibration standard, following a standard protocol.

Selected recombinant Cap preparations were additionally examined by negative-stain transmission electron microscopy (TEM) according to standard procedures to assess the formation of virus-like particles (VLPs).

### 2.5 Serum samples

Further analyses were performed using sera from pet psittacine birds that had been confirmed as BFDV-PCR-positive by the Ypelaar and/or Ogawa assay (Ogawa et al., 2005; Ypelaar et al., 1999). Sera constituted were convenience samples of residual diagnostic material and were used for proof-of-principle assessment of antibody recognition across recombinant Cap antigens. They had been collected between 2020 and 2024 and had been stored at −20 °C until analysis. A total of 28 BFDV-PCR-positive sera were initially subjected to a preliminary antibody screening using recombinant protein Cap 7, which had been selected arbitrarily as the screening antigen. Eight sera were subsequently selected based on PCR-positivity, sufficient residual volume for testing against all seven Cap variants in both Western blot and ELISA and inclusion of multiple psittacine species and collection years. In cases where the exact bird species was not known, the origin of the serum is described as “parrot”.

Sera originated from a Grey parrot (*Psittacus* sp., collection year 2024, serum 1), a galah (*Eolophus roseicapilla*, 2020, serum 2), a macaw (*Ara* hybrid, 2021, serum 3), a plain parakeet (*Brotogeris tirica*, 2021, serum 4), an Alexandrine parakeet (*Psittacula eupatria*, 2022, serum 5), an unspecified parrot (2020, serum 6), a cockatiel (*Nymphicus hollandicus*, 2024, serum 7) and another unspecified parrot (2020, serum 8). The eight birds had been naturally infected at some point in the past, but the exact time was unknown. No independent serological reference assay (e.g., HI) was performed to confirm BFDV-specific antibody status or to quantify antibody titres. Within each assay, each serum was tested at a fixed dilution against all seven Cap antigens. Analyses were therefore restricted to relative antigen-recognition patterns within individual sera, and neither absolute antibody levels nor signal intensities were compared quantitatively between birds.

### 2.6 Semi-quantitative Western blot analysis

For each recombinant Cap protein, SDS-PAGE was performed on mini gels (100 mm x 100 mm) with a total protein load of 2.5 µg per 3 mm gel width. Nitrocellulose strips measuring 3 mm in width (with an equivalent protein load of 2.5 µg per strip) were prepared by means of blotting.

A set of seven strips, each containing one of the seven recombinant Cap proteins, was incubated in parallel for each serum sample. In each experiment, an additional strip was included and incubated with anti-His tag antibody. This was involved as a quality-control measure to confirm the presence of the recombinant His-tagged Cap proteins at the expected molecular weight. Anti-His detection was not used for quantitative normalization of serum-derived signals, as it employed a separate antibody detection system and had not been validated as a quantitative loading control.

After blocking with 2 % (w/v) skimmed milk powder in PBS for 1 h at room temperature, strips were incubated overnight at 4 °C with psittacine serum diluted 1:250 in blocking buffer. Strips were washed three times for 5 min each with PBS containing 0.05 % Tween-20 (PBST) and then incubated with gentle agitation for 30 min at room temperature with an unconjugated goat anti-bird IgG (heavy and light chain) antibody (Bethyl Laboratories, Montgomery, USA, 1:200 in PBST with 2 % milk) as the secondary antibody. After a further three washes in PBST, bound goat antibodies were detected using an HRP-conjugated anti-goat IgG antibody (whole molecule, Sigma-Aldrich, 1:7500 in PBST with 2 % milk) for 30 min at room temperature with gentle agitation. Following the final wash, strips were developed using an enhanced chemiluminescence (ECL) substrate according to the manufacturer’s instructions, (Amersham ECL prime, Cytiva) and signals were recorded with a digital chemiluminescence imaging system (Celvin® S, biostep GmbH). Exposure settings were kept constant within each blot, and no automatic background correction was applied; the resulting raw images were used for subsequent analysis. Band intensities were quantified using ImageJ version 1.54 (Schneider et al., 2012). For each band, the mean grey value was measured within a standardised rectangular region of interest (ROI) of identical size as well as the mean grey value of a local background ROI adjacent to the band. Background-corrected band intensities were obtained by subtracting the local background signal from the respective Cap-specific band intensity. No negative background-corrected values occurred. To visualise relative antigen-recognition patterns within each serum, the background-corrected intensity of each Cap variant was normalised to the geometric mean of the background-corrected intensities of all seven Cap variants for that serum (see Supplementary Table S1).

### 2.7 Enzyme-linked immunosorbent assay

To determine the optimal ELISA conditions, a checkerboard titration was performed in advance of the further steps (protein concentrations 2, 4, 6, 8, 10 µg/ml), serum concentrations (1:100, 1:500; 1:1000, 1:5000, 1:10,000, 1:50,000, 1:100,000). The final conditions of 6 µg/mL antigen and a serum dilution of 1:500 were selected as the combination providing a strong specific signal while maintaining low background and adequate discrimination between reactive and negative-control sera. High-binding 96-well ELISA plates (PolySorb, Thermo Fisher Scientific) were coated overnight in carbonate-bicarbonate coating buffer (0.05 M, pH 9.6) at 4 °C with recombinant Cap protein. Plates were then washed three times with PBS containing 0.05 % Tween-20 (PBST) and blocked with 4 % (w/v) skimmed milk powder in PBST for 1 h at 37 °C. Coating efficiency was not independently quantified for the individual Cap variants. Each of the eight serum samples was diluted 1:500 in Tris-buffered saline containing 0.05 % Tween-20 (TBST) and added to antigen-coated wells in duplicate, followed by incubation for 1 h at 37 °C. After three washes with PBST, bound antibodies were detected using an HRP-conjugated goat anti-bird IgG (heavy and light chain) antibody (Bethyl Laboratories, 1:10,000 in TBST) for 1 h at 37 °C. Plates were washed again, and colour was developed with tetramethylbenzidine substrate for 10 min at room temperature in the dark. The reaction was stopped by adding 1 M H₂SO₄, and absorbance was measured at 450 nm (reference 630 nm) using a microplate reader (Dynaread, Dynex technologies, Brno, Czech Republic). Antigen-free control wells (coated with coating buffer only) and internal negative control sera from BFDV-PCR negative psittacine birds were included on each plate. The same three negative-control sera were included on each ELISA plate and were used for antigen-specific background correction and calculation of the plate-specific cut-off.

ELISAs were performed with each reaction in duplicate and in three independent assays. Duplicate absorbance values were averaged per run. For each antigen on each plate, the cut-off was defined as the mean OD of negative control sera + 3×SD (n = 6 wells derived from 3 negative sera). Consistency across runs was summarised as non-reactive (0/3), equivocal/sporadic (1/3–2/3), or reactive (3/3). For each run and antigen, background-corrected absorbance values (ΔOD) were calculated by subtracting the mean of negative sera from each measurement. Background-corrected values ≤0 were considered to represent no signal above the negative-serum background and were set to zero before calculation of proportional contributions. This transformation was used only for the proportional representation of antigen-recognition profiles. Reactivity classification was based on the original OD values relative to the antigen- and plate-specific cut-off. To visualise relative antigen recognition patterns, ΔOD values were converted to proportional contributions by dividing each antigen-specific ΔOD by the sum of ΔOD across all seven antigens for the respective serum (total = 100 %).

### 2.8 Comparison of Western blot and ELISA

Because Western blot densitometry and ELISA absorbance values differ in scale and background behaviour, results were normalised and visualised using assay-specific approaches to facilitate comparison of relative antigen recognition patterns within each assay; direct quantitative comparisons between assays were not inferred.

To assess concordance between ELISA and Western blot reactivity across the seven recombinant Cap antigens, serum-specific Spearman rank correlations were calculated using Microsoft Excel (Microsoft, Redmond, USA). For each of the eight PCR-positive sera, antigen reactivity was quantified as ELISA signal intensity (background-corrected absorbance; mean of technical replicates as described above) and Western blot signal intensity (background-corrected densitometry values/mean grey values obtained in ImageJ). The seven antigens were ranked within each serum separately for ELISA and Western blot. Because several sera produced largely similar signals across multiple antigens (i.e., limited dynamic range and frequent ties), we report ρ primarily as a descriptive measure of agreement rather than as a definitive statistical test.

## 3 Results

### 3.1 Characterisation of BFDV cap gene sequences, structural visualising of cap variability

**Fig. 1:**
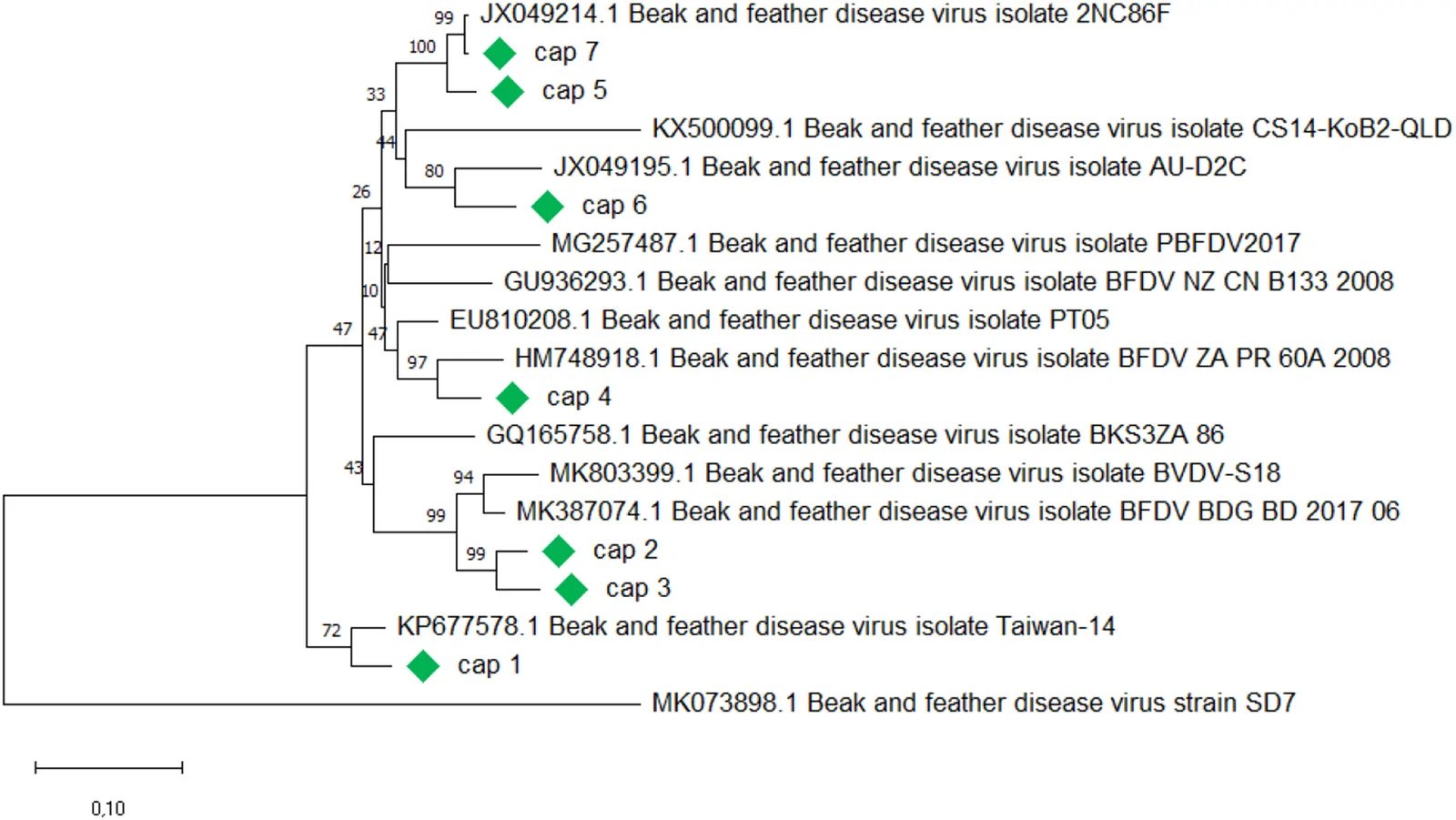
**Phylogenetic analysis of BFDV ORF2 sequences.** Maximum-likelihood tree based on a MUSCLE alignment of beak and feather disease virus (BFDV) ORF2 nucleotide sequences (735 bp), including seven sequences generated in this study (*cap* 1–7, marked with a green rhomb, GenBank accession numbers in brackets) and 12 ORF2 sequences (named acceding to their GenBank accession numbers) representing clades Ia-f and IIa-b, that were selected to reflect the genetic diversity of current BFDV genotypes (Shah et al., 2023).

**Fig. 2:**
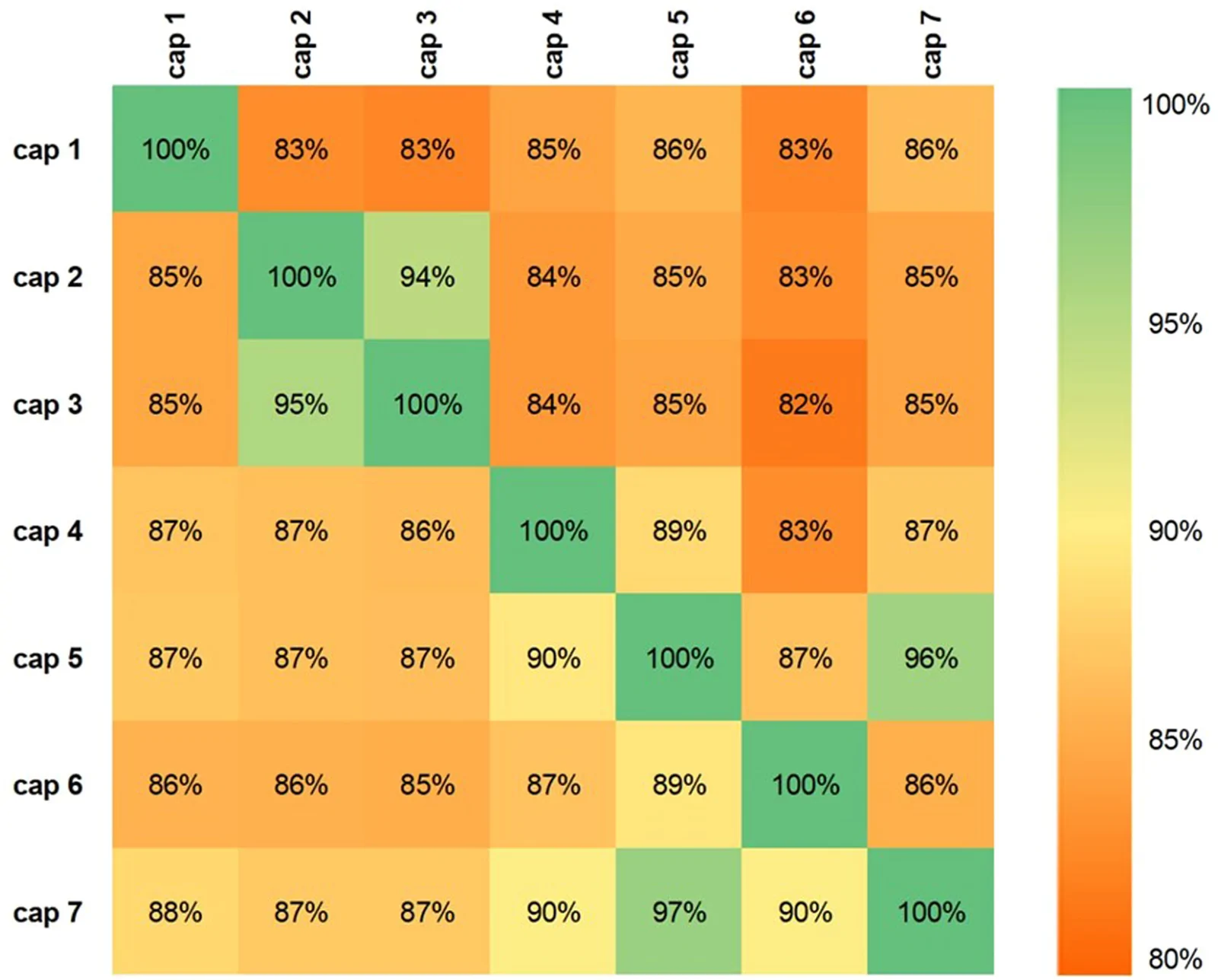
**Pairwise identity of BFDV ORF2 sequences.** Split pairwise identity matrix of seven beak and feather disease virus (BFDV) complete ORF2 sequences. The lower left triangle shows nucleotide percent identity, and the upper right triangle shows amino acid percent identity (both derived from MUSCLE alignments; % identity calculated as (1 − p-distance) × 100).

Maximum-likelihood phylogenetic analysis of seven ORF2 sequences generated in this study together with selected reference sequences from GenBank (Shah et al., 2023), confirmed that the BFDV variants included in this study cover a broad range of the BFDV diversity (Fig. 1). Across all sequences included in the analysis, pairwise ORF2 nucleotide sequence identity ranged from 65–99 % (median 87 %), and pairwise amino acid identity ranged from 64–99 % (median 84 %). Within the seven study sequences (*cap* 1-*cap* 7), pairwise nucleotide identity ranged from 85–97 % (median 87 %), and pairwise amino acid identity ranged from 82–96 % (median 85 %). These relationships are visualised as a split triangular heatmap, with nucleotide identities shown in the lower left triangle and amino acid identities in the upper right triangle (Fig. 2).

**Fig. 3:**
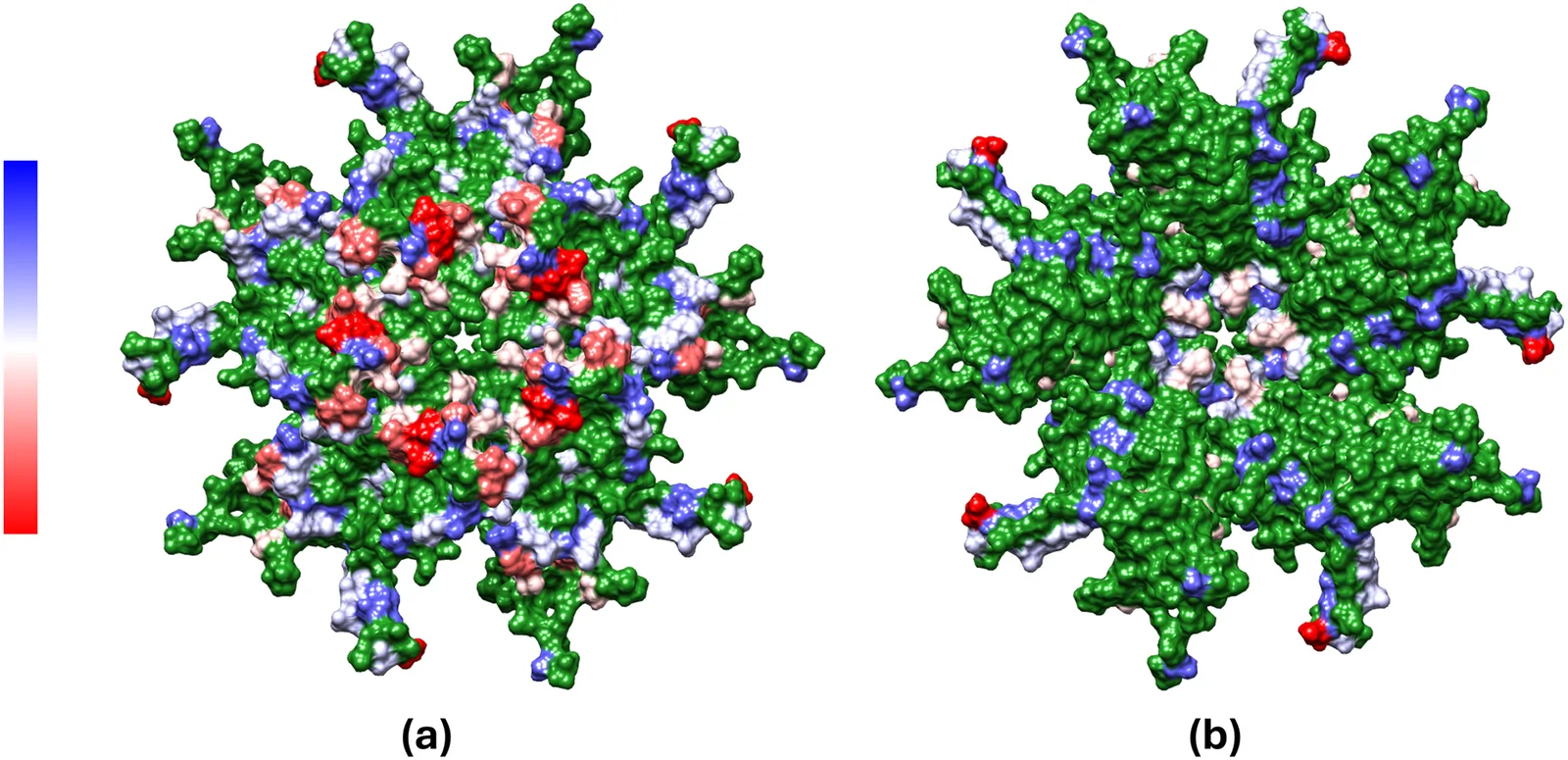
**Structural mapping of BFDV Cap variability.** Sequence variability mapped onto the surface of the BFDV capsid pentamer (PDB: 5J36). Exterior view (a) and interior view (b) of the pentameric protomer. Amino acid variability across Cap 1–7 (aligned to the 5J36 capsid sequence, full alignment shown in Supplementary Material S1) is visualized on the molecular surface using a colour gradient: highly variable positions are shown in red, more conserved positions in blue, and fully conserved residues (100 % identity across all sequences) in green.

Fig. 3 depicts sequence variability mapped onto the molecular surface of the BFDV capsid pentamer (Fig. 3a, exterior; Fig. 3b, interior), with highly variable positions shown in red, more conserved but still variable positions in blue, and fully conserved residues in green. Amino acid differences are observed across the entire pentameric surface, but variability is most prominent on the exterior face, which is also expected to contain antibody-accessible epitopes. For full amino acid alignment of all seven isolates see Supplementary Material S1.

### 3.2 Expression, purification and characterisation of recombinant Cap proteins

**Fig. 4:**
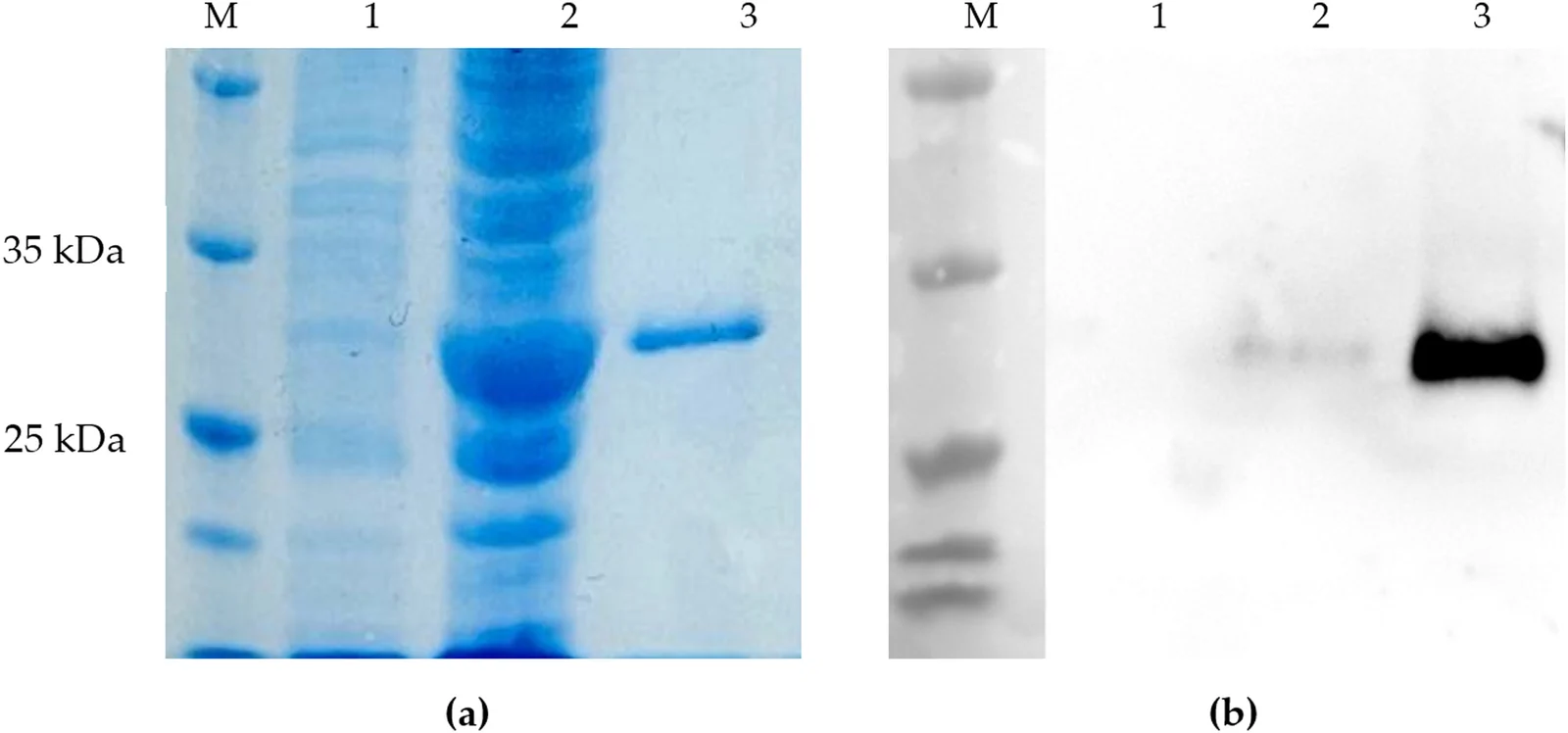
Expression and purification of recombinant Cap protein. (a) SDS-PAGE, gel stained with PageBlue™: lane M, molecular weight marker; lane 1, soluble protein from non-induced *E. coli*; lane 2, soluble protein from induced *E. coli*; lane 3, Ni-NTA–purified beak and feather disease virus (BFDV) Cap protein (elution fraction 2); (b) Western blot probed with an anti-His tag antibody: lane M, molecular weight marker; lane 1, soluble protein from non-induced *E. coli*; lane 2, soluble protein from induced *E. coli*; lane 3, Ni-NTA–purified BFDV Cap protein (elution fraction 2). Raw, uncropped images are provided in Supplementary Material S2.

**Fig. 5:**
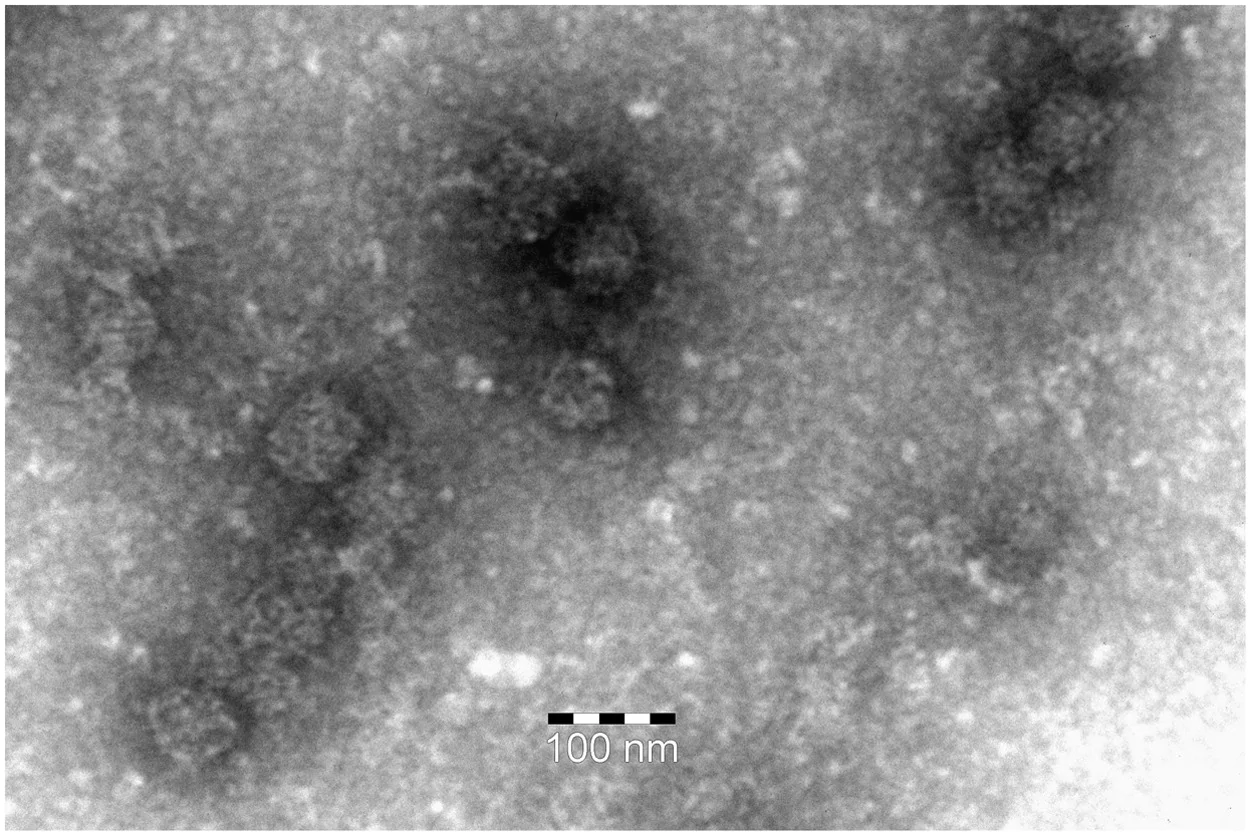
**Negative-stain TEM of recombinant BFDV Cap proteins.** Negative-stain transmission electron microscopy of selected recombinant beak and feather disease virus (BFDV) Cap preparations. Heterogeneous particulate structures and aggregates with diameters of approximately 45–85 nm are visible.

All seven N-terminally truncated Cap proteins were expressed in *E. coli* as soluble His-tagged proteins and purified by Ni-NTA affinity chromatography as shown in Fig. 4 for Cap 7 as an example. In the PageBlue-stained SDS-PAGE, a distinct band at approximately 27 kDa was absent or faint in the non-induced soluble fraction, became prominent after induction, and was strongly enriched in the elution fraction after Ni-NTA affinity chromatography for each recombinant protein (Fig. 4a). Protein identity was confirmed by anti-His Western blotting, showing specific signals at the expected molecular weight in the Ni-NTA eluates (Fig. 4b, Supplementary Material S2). Negative-stain TEM of selected truncated Cap proteins revealed heterogeneous particulate structures, and larger pleomorphic particles/aggregates (Fig. 5).

### 3.3 Semi-quantitative Western blot reactivity of sera from BFDV-positive birds

**Fig. 6:**
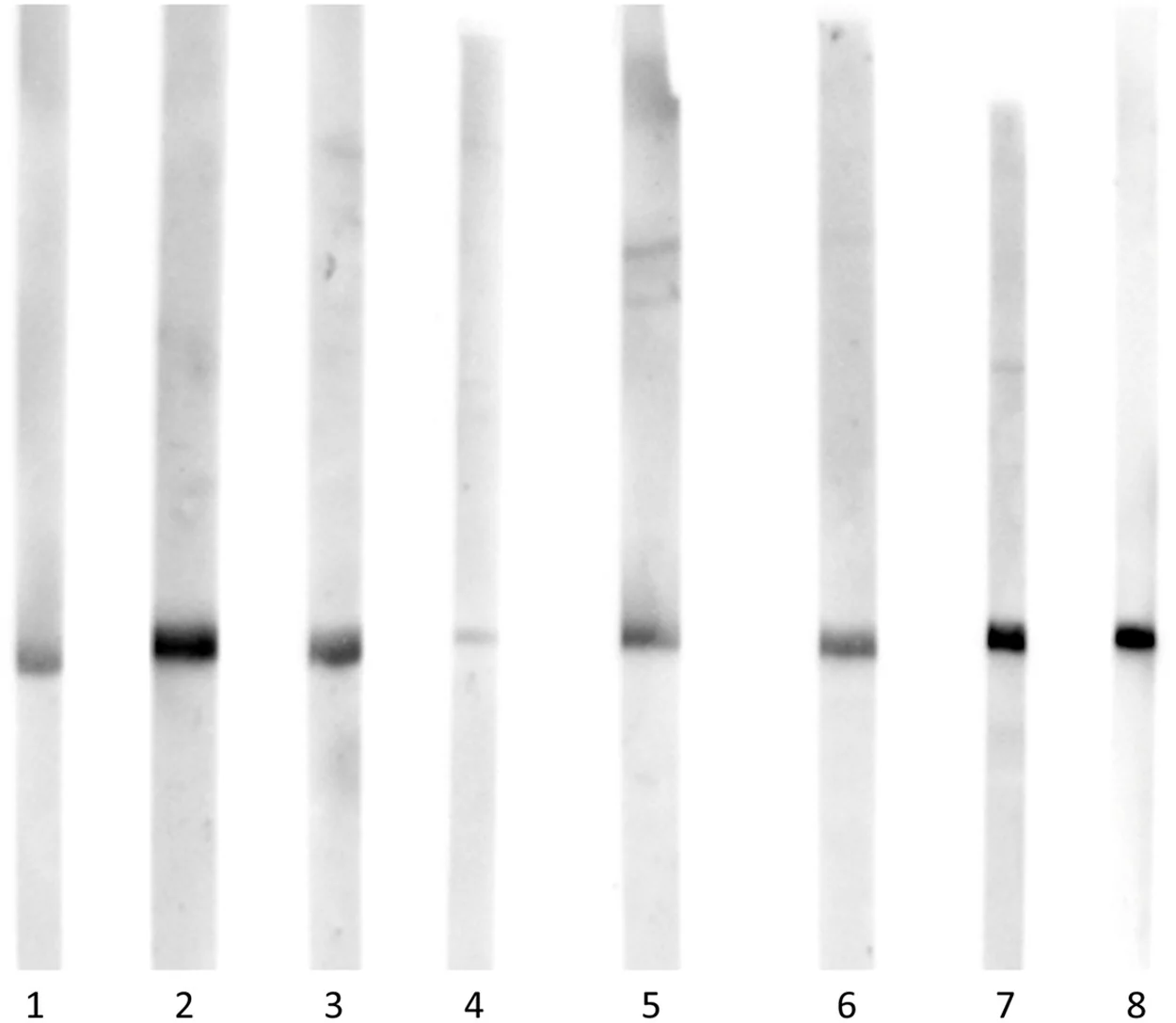
**Representative Western blot of BFDV Cap antibody reactivity.** Recombinant beak and feather disease virus (BFDV) Cap proteins Cap 1-Cap 7 (lanes 1–7) were separated by SDS-PAGE, transferred to nitrocellulose and incubated with serum from a BFDV PCR-positive hybrid macaw (serum 3, diluted 1:250), followed by goat anti-bird IgG (1:200) and HRP-conjugated anti-goat IgG (1:7500). Signals were developed using an enhanced chemiluminescence (ECL) substrate. Lane 8 shows a positive control strip containing Cap 3 probed with an anti-His-tag antibody. Raw, uncropped blots of all 8 sera are provided in Supplementary Material S3.

**Fig. 7:**
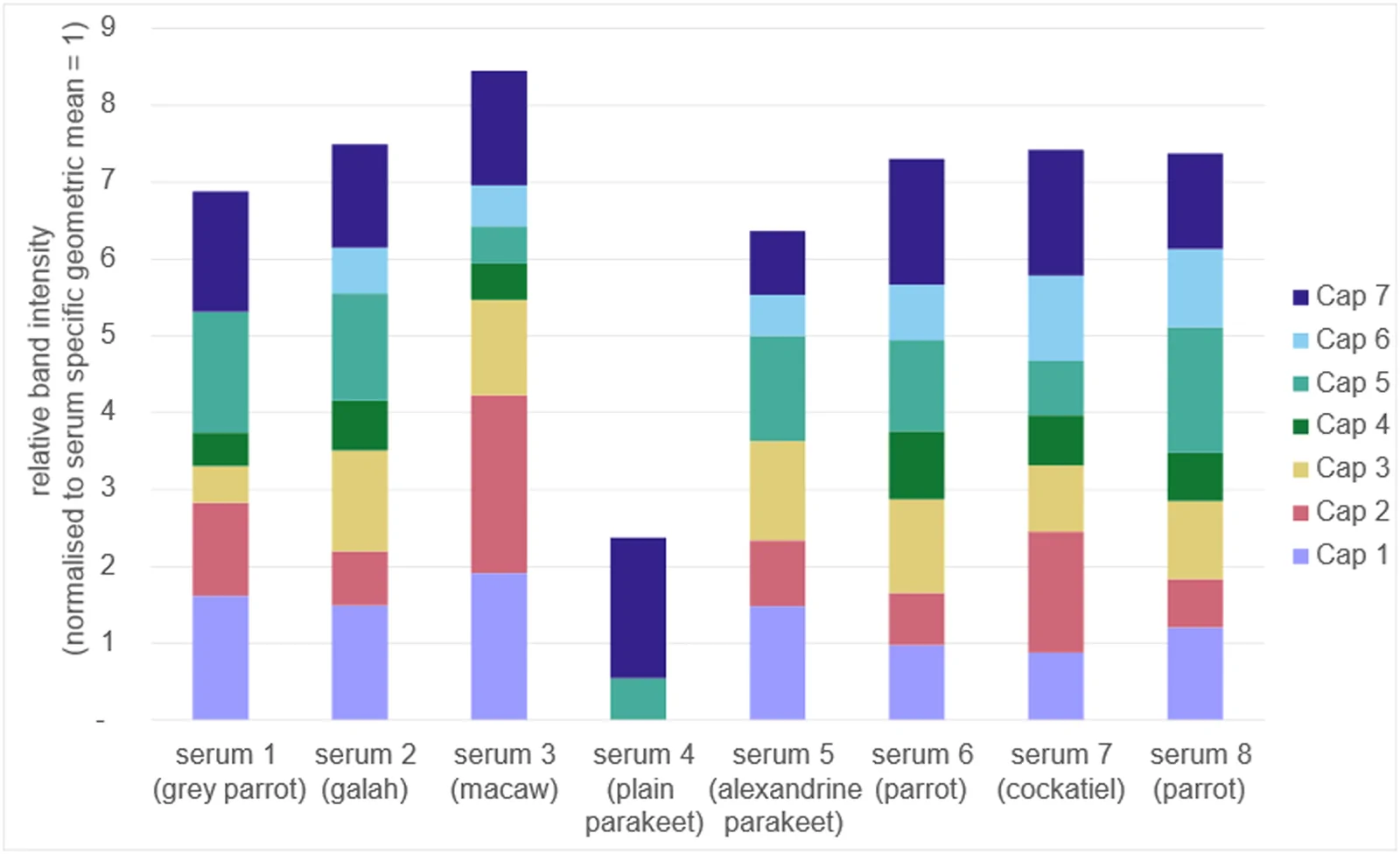
**Serum-specific Western blot reactivity against BFDV Cap variants**. Stacked bar chart summarising Western blot reactivity of eight sera against seven recombinant beak and feather disease virus (BFDV) Cap variants (Cap 1-Cap 7). Each bar represents one serum sample; coloured segments indicate the relative signal contribution of each Cap variant. Sera originated from a Grey parrot (*Psittacus* sp., serum 1), galah (*Eolophus roseicapillus*, serum 2), macaw (*Ara* hybrid, serum 3), plain parakeet (*Brotogeris tirica*, serum 4), Alexandrine parakeet (*Psittacula eupatria*, serum 5), two psittacine parrots of unspecified species (sera 6 and 8) and a cockatiel (*Nymphicus hollandicus*, serum 7). For each serum, background corrected band intensities were quantified by densitometry and normalised to the geometric mean signal across all Cap proteins (geometric mean = 1), and the plotted values represent the resulting relative (proportional) signal intensities per protein. The corresponding original and background-corrected densitometry values and raw Western blot images are provided in Supplementary Table S1 and Supplementary Material S3.

Sera from eight BFDV PCR-positive psittacines representing a broad range of species showed pronounced individual differences in Western blot reactivity across the panel of seven recombinant Cap variants (Fig. 6, Fig. 7, Supplementary Table S1, Supplementary Material S3). The pattern of relative band intensities (normalised to the geometric mean across all Cap variants) illustrated that several sera produced no visible band against some proteins under the applied exposure settings (sera 1, 4 and 5). Accordingly, using a single-antigen Western blot would have reduced sensitivity for some sera under the applied conditions. In addition, the signal intensity exhibited high variability; the Cap variant yielding the strongest signal differed between sera, whereas reactivity to other variants was weak or absent (Fig. 7).

### 3.4 ELISA reactivity across recombinant Cap variants

**Fig. 8:**
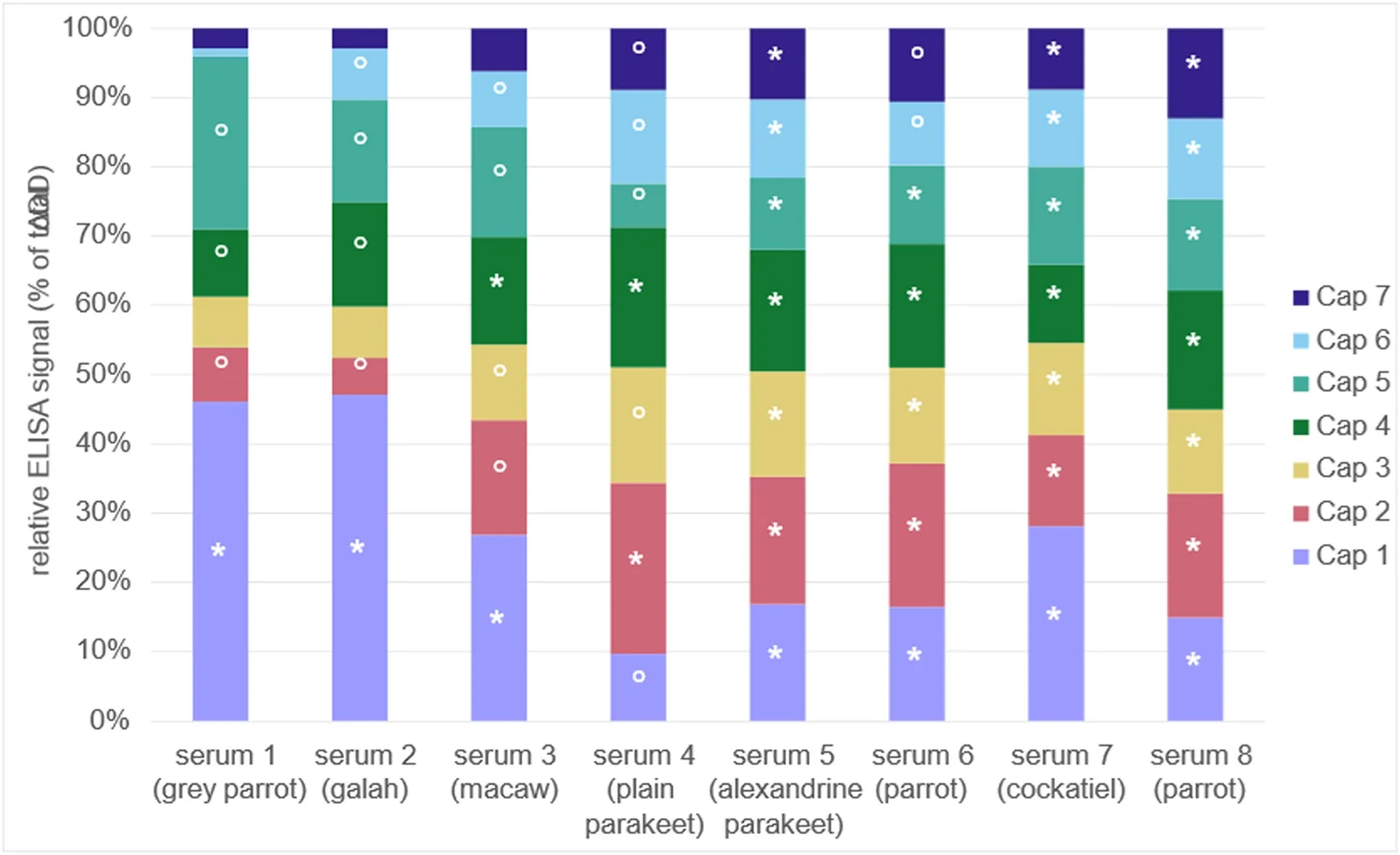
**Serum-specific ELISA reactivity profiles against BFDV Cap variants.** Normalised ELISA reactivity profiles of eight sera (origin as in Fig. 7) across seven recombinant beak and feather disease virus (BFDV) Cap variants displayed as proportional signal contributions based on background-corrected absorbance values (ΔOD; negative serum mean subtracted on a plate- and antigen-specific basis, reactivities of individual sera against the seven Cap proteins summarized to total 100 %). Asterisks (*) indicate serum-antigen combinations classified as reactive (≥ antigen-specific cut-off in 3/3 independent runs). Open circles (°) indicate equivocal/sporadic reactivity (≥ cut-off in 1/3 or 2/3 runs), whereas combinations ≥ cut-off in 0/3 runs were considered non-reactive (no symbol). Cut-offs were defined per antigen and plate as the mean of negative sera + 3×SD. Segment height reflects proportional contribution within a serum and is therefore not directly comparable to the cut-off classification, which is based on antigen-specific raw signals. Raw absorbance values from all independent ELISA experiments are provided in Supplementary Table S2.

The antigen-specific ELISA was performed in three independent assays (each in duplicates for the sera-antigen combinations). Raw absorbance values varied between runs, including differences in background signal, indicating inter-assay variation in absolute signal intensity. We aimed to compare relative signal profiles, not absolute OD values across plates analogous to the Western blot, to enable comparison of serum-specific antigen recognition patterns across experiments. Signals were background-corrected on a plate- and antigen-specific basis using representative negative sera and were subsequently expressed as proportional contributions of each Cap variant to the total ELISA signal per serum (Fig. 8, OD values shown in Supplementary Table S2).

Several serum-antigen combinations remained at or below the antigen-specific cut-off (cut-off determined as mean of three negative sera + 3 x SD), demonstrating that individual sera did not react detectably with all Cap variants under the conditions used. Conversely, sera typically showed pronounced differences in the distribution of their ELISA signal across the Cap panel, with some sera exhibiting a strong contribution from only a few variants whereas others displayed a more even pattern (Fig. 8). To account for variability near the cut-off across the three independent runs, serum-antigen combinations were categorised as reactive if they exceeded the cut-off in all three runs (3/3; *), as equivocal/sporadic if reactive in only one or two out of three runs (1/3–2/3; °), and as non-reactive if never exceeding the cut-off (0/3; no symbol) in order to avoid errors in evaluation that may be occasioned by proximity to the cut-off. As demonstrated in Fig. 7, there is a considerable variation in signal intensity, contingent on the specific antigen and serum combination.

### 3.5 Comparison of antigen recognition patterns between Western blot and ELISA

Across the eight sera, agreement between ELISA and Western blot reactivity patterns varied substantially. Serum-specific Spearman rank correlations between antigen rankings in ELISA and Western blot ranged from ρ = −0.43 to ρ = +0.64 (Supplementary Table S3), indicating that some sera showed moderate concordance (antigens ranked higher in ELISA reactivity also tended to rank higher in Western blot), whereas others displayed weak or even inverse ranking patterns. Small differences in measured signal resulted in changes in rank order, which can disproportionately affect Spearman’s ρ given the small number of antigens (n = 7). Overall, these findings support incomplete concordance between both serological readouts and suggest that, depending on the serum, ELISA and Western blot may capture partially different aspects of antibody binding to recombinant Cap variants (e.g., differences in epitope presentation and assay format), with the greatest instability occurring when signal strengths cluster within a narrow range.

Across the full dataset, only two Cap variants (Cap 5 and Cap 7) produced a detectable band in the Western blot for all eight sera. In the ELISA, no individual Cap variant was reactive in all sera under the applied cut-off criteria. Conversely, five sera (2, 3, 6, 7 and 8) showed reactivity against all seven Cap variants in the Western blot, whereas three sera (5, 7 and 8) were reactive against all seven Cap variants in the ELISA. When the Cap panel was considered collectively, all sera showed anti-Cap reactivity to at least one variant. However, when individual Cap variants were evaluated separately, reactivity was incomplete and strongly dependent on the specific serum-antigen combination. Accordingly, under the conditions applied here, some sera would have been classified as non-reactive if only a single Cap variant had been used, despite showing detectable antibody binding to alternative Cap variants within the same assay panel. For the samples tested in the present study, this could increase the likelihood of a false-negative serological classification in a diagnostic assay relied on only a single Cap antigen, particularly for samples with low or borderline reactivity.

## 4 Discussion

In this study, we evaluated the serological reactivity of psittacine sera against a panel of seven recombinant antigens representing genetically diverse BFDV capsid variants using ELISA and Western blot. To our knowledge, this is the first systematic BFDV study to assess a panel of recombinant Cap variants against multiple sera in parallel, providing a comparative view of antigen-serum specific reactivity and diversity.

The seven recombinant Cap proteins were tested against sera of eight BFDV PCR-positive psittacines which had been kept as pet birds and tested in routine diagnostics revealing natural BFDV infections. We observed clear antigen-serum dependent differences in seroreactivity across the panel. These findings confirm our research hypothesis demonstrating that genetically diverse Cap variants can display different antibody-binding profiles and that sequence variation in ORF2 could translate into differences in epitope composition and/or presentation of the capsid protein. The marked differences in reactivity among the seven antigens suggest that antibody binding is not uniformly conserved across genetically diverse capsid variants. A plausible explanation is variation in the availability and conformation of epitopes between variants; even limited amino acid differences within key epitopes may disproportionately affect binding. These differences in antibody binding between Cap variants may be relevant for immune responses during natural infection and for the performance of Cap-based vaccines. Moreover, these findings indicate that antigen selection can influence the detection of antibody binding in Cap-based serological assays.

In selecting Cap variants for this panel, we aimed to capture the diversity of BFDV genotypes by including both closely related and more divergent ORF2 sequences. Sequence selection was guided by a GenBank reference set according to Shah et al. (Shah et al., 2023). Our phylogenetic analyses indicate that the seven study sequences fall within and nearly span the range of BFDV ORF2 diversity of the described BFDV clades.

For circoviruses, a threshold of 80 % genome-wide nucleotide identity has been proposed as a species demarcation criterion (ICTV, 2026a), but there is not an officially accepted ICTV (International Committee on Taxonomy of Viruses) scheme for defining genotypes or subtypes within BFDV. Several studies have attempted to structure this diversity more finely, proposing strain and subtype thresholds or phylogenetic clades and sub-clades and reporting clusters that are partly host- or region-associated (Heath et al., 2004; Shah et al., 2023; Varsani et al., 2011). These efforts illustrate that BFDV is genetically diverse enough to warrant more detailed classification, but they have not yet converged on a unified nomenclature. Within our dataset, pairwise ORF2 identities were in median ∼86 % at the nucleotide level, with even greater divergence at the amino acid level, placing the variants in a range where distinct strain or subtype categories would be plausible under previously suggested criteria. Sequence divergence does not necessarily translate into a linear change in seroreactivity, but in this study closely related variants showed similar antibody-binding patterns in some cases. Most notably, Cap 5 and Cap 7 shared the highest amino acid sequence identity among the investigated variants (96 %), and serum 4 reacted exclusively with these two variants in Western blot. The genetic diversity provides a coherent framework for interpreting the heterogeneous antibody binding patterns observed in this study.

Comparable, although somewhat more homogeneous, pairwise ORF2 identities have been reported for porcine circovirus type 2 (PCV2, species Circovirus porcine2) genotypes. In a dataset comprising PCV2a, PCV2b and PCV2d isolates, ORF2 nucleotide and amino acid identities across all sequences ranged from 89.7–100 % and 88.5–100 %, respectively, whereas pairwise identities within individual genotypes were substantially higher, typically around 95–100 % (Lekcharoensuk et al., 2004; Wei et al., 2019). These values overlap with those observed for our BFDV ORF2 panel, which shows even more sequence diversity. Together with ORF2-based genotyping frameworks for PCV2, this suggests that a similarly standardised intraspecies classification scheme could also be beneficial for BFDV, particularly if it were explicitly linked to antigenic properties of different Cap variants rather than relying solely on sequence-based thresholds.

For PCV2 the serological consequences of capsid variation have been characterised in considerably more detail. Epitope-mapping studies using PCV1/PCV2 chimeras and site-directed mutants have shown that even single amino acid substitutions in exposed regions of Cap can abrogate binding of neutralising monoclonal antibodies and alter virus neutralisation patterns (Lekcharoensuk et al., 2004; Shang et al., 2009). Cross-neutralisation and vaccine studies further demonstrate that PCV2 genotypes differ antigenically and that Cap antigens derived from certain genotypes (e.g., PCV2d) provide broader heterologous protection than others (Kang et al., 2020, 2021). For BFDV, Ho et al. (2018) identified a conserved linear Cap epitope (NFEDYRI) recognised by a monoclonal anti-Cap antibody. This epitope was completely conserved among all seven Cap variants investigated in the present study. Nevertheless, pronounced differences in antibody reactivity were observed between the variants, indicating that sequence variation outside this specified epitope may be responsible for the heterogeneous binding patterns observed with the polyclonal field sera. In contrast to PCV, only limited evidence exists for antigenically distinct isolates for BFDV (Shearer et al., 2008b, 2008a), and systematic analyses linking defined Cap sequence variation to antibody binding profiles or protection were largely lacking, a gap that the present study addressed.

In our study, comparison of ELISA and Western blot results revealed partial concordance but also systematic discordances, particularly for serum samples with antibody binding levels close to the decision thresholds. These differences cannot be attributed to a single factor, as Western blot and ELISA differ substantially in their analytical principles and experimental conditions. Western blotting is performed under denaturing conditions and predominantly detects antibodies to linear epitopes after denaturation, whereas ELISA may retain, alter or mask conformational and linear epitopes depending on folding and coating conditions, and conformational epitopes may mask or modify access to linear epitopes (Forsström et al., 2015). In addition, the assays differed in serum dilution, antibody detection systems, signal generation and dynamic range, which may influence relative signal intensities and antigen rankings. Consequently, the observed discordance between Western blot and ELISA likely reflects a combination of differences in epitope accessibility, antigen presentation and assay-specific analytical characteristics. Furthermore, background signal and non-specific binding represent a major limitation in immunoassays and directly affect discrimination around the cut-off. We applied local background correction and plate-specific cut-offs to reduce systematic bias, yet several sera produced broadly similar signal intensities across multiple antigens in one or both assays. Under these conditions, small measurement differences can substantially alter rank order, leading to variable Spearman correlation coefficients and borderline classifications. Both tests, however, revealed differences in antigen-binding patterns among the sera included in our study thus confirming our research hypothesis.

Earlier serological work using haemagglutination (HA) and haemagglutination inhibition (HI) assays largely failed to identify distinct BFDV serotypes. In cross-reactivity experiments with multiple isolates and high-titre sera from different psittacine species, no consistent evidence for antigenically discrete serotypes was found and broad HI cross-reactivity was reported (Khalesi et al., 2005; Raidal et al., 2008). However, a subsequent study on cockatiels described a BFDV isolate that was not inhibited by all high-titre sera tested and was interpreted as antigenically distinct from previously characterised viruses, potentially representing a separate serotype (Shearer et al., 2008a, 2008b). Taken together, these data suggested substantial antigenic homology among many BFDV genotypes while leaving open the possibility that more divergent variants may differ antigenically and form antigenic subgroups. Our data demonstrate distinguishable antibody-binding profiles among genetically diverse BFDV Cap variants, although the extent to which these differences are quantitatively related to amino acid sequence divergence remains to be determined. This is compatible with the possibility of antigenic subgroups existing within BFDV, even if they are not yet formally defined as serotypes.

The genetic and serological diversity documented here has implications for our understanding of the dynamics of natural BFDV infections. Mixed infections with multiple BFDV variants in individual birds have been documented (Ogawa et al., 2013; Sarker et al., 2014). Incomplete or absent cross-protection is a plausible explanation for secondary infections with more distant genotypes, but no controlled in vivo data are currently available to confirm or refute this. Furthermore, presumed immunosuppression due to a pre-existing BFDV infection must also be considered in this context.

A limited number of experimental PBFD vaccination studies in psittacines has been published so far. They have used inactivated whole virus or a single recombinant Cap antigen and have typically challenged birds with the same or a closely related virus isolate (Bonne et al., 2009; Regnard, 2015; Shearer et al., 2009). A range of Cap- and VLP-based BFDV vaccine candidates have been shown to induce seroconversion and robust antibody responses in experimental settings. However, available challenge studies indicated that protection was incomplete, with vaccination primarily reducing clinical disease and virus shedding rather than preventing infection altogether (Bonne et al., 2009; Regnard, 2015). For several newer VLP formulations, protective efficacy has not yet been assessed in controlled challenge experiments, and data on the duration of immunity and on cross-protection against genetically divergent BFDV variants remain very limited (Das et al., 2026; Mulondo et al., 2025). Genetic relationships between vaccine antigens and challenge viruses were not reported in detail, and potential effects of sequence mismatches on vaccine efficacy have not been systematically addressed. Although variation in antibody recognition may be relevant when selecting Cap antigens for future vaccine studies, the present data do not provide information on immunogenicity, neutralising activity, or protective efficacy. Consequently, there are currently no robust data to define the degree of sequence divergence in Cap that can be tolerated before vaccine-induced protection is compromised. In addition, when protein-based vaccines are used, the effects of post-translational modifications, which differ in various protein expression systems, must be considered. At present, in the absence of a licensed BFDV vaccine, prevention of spread relies on the identification of infected birds and their separation from uninfected individuals or flocks. Reliable diagnostics and high analytical sensitivity are therefore central to disease control. Our study was not designed as a formal test validation, and the sample size is too small to establish assay performance parameters. Rather, it was adapted to examine how genetically diverse Cap variants interact with different sera. Within this framework and as mentioned above, in both assays some serum-antigen pairs remained at or below the antigen-specific cut-off, so that a single recombinant Cap antigen would have failed to identify sera of certain PCR-positive birds as reactive under the conditions used. In most published BFDV serological assays, a single recombinant Cap is used as antigen (Ho et al., 2018; Shearer et al., 2009) which is, according to our results, associated with a risk of false classification in serological assays. In contrast our data argue for one of the three following strategies: single-antigen coating using a variant with the most consistent reactivity across a large validation panel; mixed-antigen coating using a small number of genetically divergent representatives (e.g. covering major clades) to increase breadth; or parallel coating (multi-antigen format). As suggested for other viruses, e.g. highly pathogenic avian influenza virus H5 (Kok et al., 2025), it may be possible to identify a Cap antigen located centrally within the BFDV antigenic space and to use it for both vaccination and diagnostics. Antigenic mapping could be a useful tool for such future investigations. Multi-antigen strategies can be used, where panels of antigens are combined to increase detection range and could be applied to BFDV to relieve antigen-specific blind spots. In our limited dataset, combinations of Cap variants would have been sufficient to detect all eight sera as reactive, while a single antigen would not. Given the exploratory nature of our study and the small number of sera used, such combinations can only be regarded as illustrative rather than prescriptive and we cannot recommend or favour any optimal testing strategy at this time. Nevertheless our findings highlight that antigen selection can have a major impact on apparent sensitivity, and that rationally designed antigen panels might improve the robustness of BFDV serology under field conditions. Further investigations are needed at this point.

Expression of large amounts of purified Cap has previously been reported to be troubled by insolubility and aggregation, with improved yields obtained using truncated (Johne et al., 2004; Regnard, 2015) and codon-optimised constructs. Consistent with this, initial attempts to express full-length Cap in our system resulted in solubility issues, so we used N-terminally truncated, codon-optimised Cap variants lacking 40 amino acids. This strategy yielded high amounts of soluble recombinant protein. In line with reports from other small icosahedral viruses, where N-terminal truncations can shift the assembly towards alternative T-numbers and particle sizes (Mo et al., 2019; Yang et al., 2013), negative-stain transmission electron microscopy in our study revealed heterogeneous particulate structures that were markedly larger (approximately 45–85 nm) and frequently aggregated, rather than forming a homogeneous population of particles in the size range expected for native circoviruses (∼15–20 nm). These observations suggest aberrant assembly or aggregation. Such deviations have been described for misfolded or partially assembled viral capsid proteins in other systems (Bhat et al., 2022; Mejía-Méndez et al., 2022). Despite the truncated cap proteins not being equivalent to the native BFDV cap protein, the recombinant Cap variants produced robust signals with both anti-His antibodies and field sera, indicating that they retained relevant epitopes for antibody binding despite their altered appearance.

### 4.1 Limitations

Several limitations of the present study should be considered. First, the number of sera and Cap variants was limited, and all sera were derived from PCR-positive birds with unknown infection histories and immune status, which constrains generalisability. In addition, the preliminary serum screening was performed using Cap 7 only. The use of a single antigen for preliminary screening represents a potential source of selection bias that should be considered when interpreting the particularly broad reactivity observed for this variant. Furthermore, the ORF2 sequences of the BFDV strains infecting the serum donors were not available. Consequently, homologous versus heterologous antibody recognition could not be assessed, and it remains unknown whether stronger reactivity towards individual Cap variants reflected closer genetic relationship to the respective infecting strain. Despite the small sample size and the unknown clinical background of the birds, we still observed broad differences between Cap variants and were thus able to confirm our research hypothesis.

Second, only N-terminally truncated cap constructs were examined in this study. Although truncated Cap proteins previously have been used for BFDV serological studies, deletion of the N-terminal region may affect protein folding and capsid assembly and thereby alter the presentation of conformational and linear epitopes. Consequently, the antibody-binding patterns observed with the recombinant proteins cannot be assumed to fully reproduce those directed against native full-length Cap. Furthermore, an expression system in *E. coli* was used, as expression systems for recombinant Cap in avian cells and routine virus cultivation for BFDV have not yet been commercially established. In addition, although equal total protein amounts were used for the individual Cap variants, this does not necessarily ensure equal amounts of intact Cap protein in the Western blot or equal amounts of immobilized antigen in the ELISA. Therefore, differences in the effective amount of antigen available for antibody binding cannot be completely excluded as a contributor to the observed variation in signal intensity. Despite the favourable outcomes observed in protein yield, protein staining, Western blot and ELISA experiments, the results must be interpreted with caution.

Third, we did not determine antibody titres using an independent serological reference method (e.g. HI). In addition, because a general anti-bird secondary antibody was used, differences in detection efficiency between psittacine species cannot be fully excluded. Importantly, each serum was tested at a constant dilution against all seven Cap antigens within each assay, as the present study focused on comparing relative signal intensities across different Cap antigens within the same serum, rather than comparing absolute reactivity between sera or species. Differences in overall antibody titres may nevertheless have influenced absolute signal intensities and particularly reactions close to the detection thresholds and these aspects should be addressed in larger, better-characterised validation panels. Finally, we did not assess functional antibody activities such as virus neutralisation or in vivo protection, so our observations relate to binding reactivity rather than protective immunity. The impact of these findings on vaccination strategies and cross-protectivity must be demonstrated in future challenge trials.

### 4.2 Conclusions

The current data demonstrate, within individual sera, heterogeneous antibody binding among genetically diverse BFDV Cap variants in both Western Blot and ELISA formats. These findings indicate that antigen selection can influence antibody detection in Cap-based serological assays and provide a basis for the consideration of viral diversity explicitly in the design of serological assays and future vaccines. They also offer a framework for future studies linking natural infection dynamics and pathogenesis to antigenic diversity of BFDV in psittacine hosts.

## Ethical approval

The study followed institutional and national standards for the care and use of animals. It was approved by the Ethics Committee of the Veterinary Faculty, LMU Munch (262–09–03–2021).

## Funding

This research received no external funding.

## CRediT authorship contribution statement

**Elora Kellerstrass:** Writing – review & editing, Writing – original draft, Methodology, Investigation, Data curation, Conceptualization. **Monika Rinder:** Writing – review & editing, Supervision, Methodology, Conceptualization.

## Declaration of competing interest

The authors declare that they have no known competing financial interests or personal relationships that could have appeared to influence the work reported in this paper.

## Data Availability

The nucleotide sequences of the full cap genes obtained from the seven BFDV genotypes in this study are available on the NCBI GenBank database (https://www.ncbi.nlm.nih.gov/genbank/) under the accession numbers PPX789812-PX789818. All other data generated or analysed during this study are included in this article or will be made available on request.

## Appendix Supplementary materials

Image, application 1

Image, application 2

Image, application 3

Image, application 4

Image, application 5

Image, application 6
