# Supplementary material for: Beak and feather disease virus variant-dependent antibody reactivity in naturally infected psittacine birds
Source: Virus Res. 2026 Aug 16;372:199791. doi: 10.1016/j.virusres.2026.199791 (PMC13521223; doi:10.1016/j.virusres.2026.199791)
Supplement: Supplementary file 1 [file mmc1.docx]

Supplementary Material S2a

1. **SDS- Page performed in duplicate from left to right:**

Lane 1,6: molecular weight marker

Lane 2,7: soluble protein from non-induced *E. coli*

Lane 3,8: soluble protein from induced *E. coli*

Lane 4,9: Ni-NTA purified beak and feather disease virus Cap 7 protein (elution fraction 2)

Lane 5,10: other known His-tagged recombinant protein (N-protein, bornavirus) for reference

2. **Western Blot probed with anti-His tag antibody from left to right:**

Lane 1: molecular weight marker

Lane 2: soluble protein from non-induced *E. coli*

Lane 3: soluble protein from induced *E. coli*

Lane 4: Ni-NTA purified beak and feather disease virus Cap 7 protein (elution fraction 2)

Lane 5: other known His-tagged recombinant protein (N-protein, bornavirus) for reference
