# Supplementary material for: Beak and feather disease virus variant-dependent antibody reactivity in naturally infected psittacine birds
Source: Virus Res. 2026 Aug 16;372:199791. doi: 10.1016/j.virusres.2026.199791 (PMC13521223; doi:10.1016/j.virusres.2026.199791)
Supplement: Supplementary file 2 [file mmc3.docx]

Supplementary Material S3

**Image 1: Serum 1**

From left to right: recombinant beak and feather disease virus (BFDV) Cap proteins Cap 1-Cap 7 (lanes 1-7), anti-His tag control (lane 8)

**Image 2: Serum 2**

From left to right: recombinant beak and feather disease virus (BFDV) Cap proteins Cap 1-Cap 7 (lanes 1-7), anti-His tag control (lane 8)

**Image 3: Serum 3**

From left to right: recombinant beak and feather disease virus (BFDV): lane 1 – Cap 1, lane 2 – Cap 2, lane 3 – Cap 3, lane 4 -Cap 4, lane 5 – Cap 5, lane 6 – Cap 6 (other set of strips, not used for measurements), lane 7 – Cap 7, lane 8 – Cap 3 (other set of strips, not used for measurements), lane 9 – Cap 6, anti-His tag control (lane 10)

**Image 4: Serum 4**

From left to right: recombinant beak and feather disease virus (BFDV) Cap proteins Cap 1-Cap 7 (lanes 1-7), anti-His tag control (lane 8)

**Image 5: Serum 5**

From left to right: recombinant beak and feather disease virus (BFDV) Cap proteins Cap 1-Cap 7 (lanes 1-7), anti-His tag control (lane 8)

**Image 6: Serum 6**

From left to right: recombinant beak and feather disease virus (BFDV) Cap proteins Cap 1-Cap 7 (lanes 1-7), anti-His tag control (lane 8)

**Image 7: Serum 7**

From left to right: recombinant beak and feather disease virus (BFDV) Cap proteins Cap 1-Cap 7 (lanes 1-7), anti-His tag control (lane 8)

**Image 8: Serum 8**

From left to right: recombinant beak and feather disease virus (BFDV) Cap proteins Cap 1-Cap 7 (lanes 1-7, lane 1 placed upside down)
